# Microbial Consortium of PGPR, Rhizobia and Arbuscular Mycorrhizal Fungus Makes Pea Mutant SGECd^t^ Comparable with Indian Mustard in Cadmium Tolerance and Accumulation

**DOI:** 10.3390/plants9080975

**Published:** 2020-07-31

**Authors:** Andrey A. Belimov, Alexander I. Shaposhnikov, Tatiana S. Azarova, Natalia M. Makarova, Vera I. Safronova, Vladimir A. Litvinskiy, Vladimir V. Nosikov, Aleksey A. Zavalin, Igor A. Tikhonovich

**Affiliations:** 1 All-Russia Research Institute for Agricultural Microbiology, Podbelskogo sh. 3, Pushkin, 196608 Saint-Petersburg, Russia; ai-shaposhnikov@mail.ru (A.I.S.); tatjana-aza@yandex.ru (T.S.A.); n.m.46@yandex.ru (N.M.M.); v.safronova@rambler.ru (V.I.S.); arriam2008@yandex.ru (I.A.T.); 2 Pryanishnikov Institute of Agrochemisty, Pryanishnikova str. 31A, 127434 Moscow, Russia; vl.litvinsky@gmail.com (V.A.L.); vniiasekr@yandex.ru (V.V.N.); otdzem@mail.ru (A.A.Z.); 3 Saint-Petersburg State University, University Embankment, 199034 Saint-Petersburg, Russia

**Keywords:** heavy metals, mycorrhiza, nodulation, pea, PGPR, phytoremediation

## Abstract

Cadmium (Cd) is one of the most widespread and toxic soil pollutants that inhibits plant growth and microbial activity. Polluted soils can be remediated using plants that either accumulate metals (phytoextraction) or convert them to biologically inaccessible forms (phytostabilization). The phytoremediation potential of a symbiotic system comprising the Cd-tolerant pea (*Pisum sativum* L.) mutant SGECd^t^ and selected Cd-tolerant microorganisms, such as plant growth-promoting rhizobacterium *Variovorax paradoxus* 5C-2, nodule bacterium *Rhizobium leguminosarum* bv. *viciae* RCAM1066, and arbuscular mycorrhizal fungus *Glomus* sp. 1Fo, was evaluated in comparison with wild-type pea SGE and the Cd-accumulating plant Indian mustard (*Brassica juncea* L. Czern.) VIR263. Plants were grown in pots in sterilized uncontaminated or Cd-supplemented (15 mg Cd kg^−1^) soil and inoculated or not with the microbial consortium. Cadmium significantly inhibited growth of uninoculated and particularly inoculated SGE plants, but had no effect on SGECd^t^ and decreased shoot biomass of *B. juncea*. Inoculation with the microbial consortium more than doubled pea biomass (both genotypes) irrespective of Cd contamination, but had little effect on *B. juncea* biomass. Cadmium decreased nodule number and acetylene reduction activity of SGE by 5.6 and 10.8 times, whereas this decrease in SGECd^t^ was 2.1 and 2.8 times only, and the frequency of mycorrhizal structures decreased only in SGE roots. Inoculation decreased shoot Cd concentration and increased seed Cd concentration of both pea genotypes, but had little effect on Cd concentration of *B. juncea*. Inoculation also significantly increased concentration and/or accumulation of nutrients (Ca, Fe, K, Mg, Mn, N, P, S, and Zn) by Cd-treated pea plants, particularly by the SGECd^t^ mutant. Shoot Cd concentration of SGECd^t^ was twice that of SGE, and the inoculated SGECd^t^ had approximately similar Cd accumulation capacity as compared with *B. juncea*. Thus, plant–microbe systems based on Cd-tolerant micro-symbionts and plant genotypes offer considerable opportunities to increase plant HM tolerance and accumulation.

## 1. Introduction

Most of the commonly known plants recommended for phytoremediation of heavy metal (HM) polluted soils belong to the family Brassicaceae, because of their relatively high metal tolerance and accumulation [1,2,3]. Species such as the agricultural crop Indian mustard (*Brassica juncea* (L.) Czern & Coss) have lower HM tolerance than hyperaccumulators but produce more biomass and thus extract more HMs. Although HM-hyperaccumulating species (e.g., *Thlaspi caerulescens* J.Presl & C.Presl) are very HM-tolerant, their low growth rate and biomass limit their utility for phytoremediation of new sites. This has stimulated the search for suitable plants of other families, including legumes (family Fabaceae), which grow rapidly to produce high biomass but are characterized by relatively low tolerance to HMs [4,5] and translocation of HMs from roots to shoots [6,7].

Unlike representatives of Brassicaceae, legumes form nitrogen-fixing symbiosis with nodule bacteria (rhizobia) and obligate symbiosis with arbuscular mycorrhizal fungi (AMF), which mainly supply the plant with nitrogen and phosphorus, respectively. In addition, associative symbiosis of legumes with plant growth-promoting rhizobacteria (PGPR) exerts multiple beneficial effects on plant growth and nutrition. An advanced symbiotic potential of legumes helps improve soil fertility, biodiversity and activity of soil biota, soil genesis and hence maintains and restores healthy ecosystems. Symbiotic interactions of legumes with rhizobia [8,9], AMF [10,11,12,13], and PGPR [11,14,15,16,17] substantially increased legume adaptation to abiotic stresses, potentially allowing these technologies to be used to remediate arid and contaminated soils. While these interactions have been discussed in detail [18], of particular note is the important role of the bacterial enzyme 1-aminocyclopropane-1-carboxylate (ACC) deaminase in increasing the resistance of plants, including legumes, to HMs by decreasing biosynthesis of stress phytohormone ethylene [19,20,21].

Additive and synergistic effects on plant growth and nutrition, as well as positive interactions between microorganisms, were achieved when legume plants were inoculated with various combinations of the abovementioned symbiotic microorganisms [22,23,24]. However, there are few reports of combined inoculations with micro-symbionts enhancing adaptation of legumes to HM stress. When *Trifolium repens* was cultivated in Cd-supplemented soil, co-inoculation with *Glomus mosseae* and *Brevibacillus brevis* had additive effects on plant growth, accumulation of nutrient elements and toxic Cd [25]. Moreover, *B. brevis* stimulated nodulation on roots by native rhizobia present in soil, probably due to the indole acetic acid produced by the bacterium [26]. Similar results were obtained with *Glomus mosseae* and PGPR strain *Bacillus cereus* [27]. Synergistic increase in shoot biomass, nodulation frequency and accumulation of Cd, Pb, and Zn, along with better uptake of nutrients (*n*, P, K, Ca, Mg, and Fe), was observed in *Lotus edulis* plants cultivated in HM-contaminated soil and inoculated with a mixture of *Mesorhizobium loti* and *Variovorax paradoxus* [28]. Combined inoculation with *Sinorhizobium meliloti* and *Paenibacillus mucilaginosus* improved growth and increased shoot Cu concentration, but decreased oxidative stress in *Medicago lupulina* plants grown in Cu-contaminated soil [29]. Synergistic effect on decrease in Cd, Cu, and Zn concentrations was observed in *Lupinus albus* plants inoculated with *Bradyrhizobium lupini*, *Ochrobactrum* sp. and *Pseudomonas* sp. [30]. Since polluted sites often contain a mixture of toxic metals and are subjected to other stresses (aridity, low nutrients, erosion, and extreme pH values), applying microbial consortia with multiple complementary beneficial traits may improve the phytoremediation processes.

Selecting resistant microbes and plants is needed for effective symbiotic function in the presence of toxic metals because legumes are relatively sensitive to heavy metals. Although plant mutants with altered metal tolerance and accumulation have been used to study physiological mechanisms regulating these traits [31,32,33], using these plants to study the role of microorganisms in plant–HMs interactions and phytoremediation of polluted soils is very limited. In this respect, the pea (*Pisum sativum* L.) mutant SGECd^t^ with increased Cd tolerance and Cd accumulation [34] is of particular interest. This mutant maintained better growth, nutrient homeostasis [34,35], water relations [36], and root function [37] than the wild-type SGE line when grown in Cd-containing nutrient solutions. Inoculation with the PGPR *V. paradoxus* strain 5C-2 containing ACC deaminase alleviated the negative effect of Cd on nodulation and nitrogen fixation in SGECd^t^, suggesting that plant genotype mediates the legume–rhizobia symbiosis [38]. Recently rhizobial ACC deaminase of *Rhizobium leguminosarum* bv. *viciae* strain RCAM1066 was shown to increase nodulation, nutrient uptake, and Cd tolerance of SGECd^t^ grown in Cd-supplemented soil [39].

This report aimed to investigate a beneficial role of plant–microbe interactions for tolerance of legume plants to toxic Cd and to evaluate the phytoremediation potential of a symbiotic system comprising the Cd-tolerant pea mutant SGECd^t^ and a consortium of previously selected Cd-tolerant microorganisms (PGPR *V. paradoxus*, nodule bacterium *R. leguminosarum* bv. *Viciae*, and AMF *Glomus* sp.). It was compared with wild-type plants and the Indian mustard (*Brassica juncea*) plant, which is actively used in phytoremediation [1,2,3].

## 2. Results

### 2.1. Plant Growth Parameters

**Figure 1:**
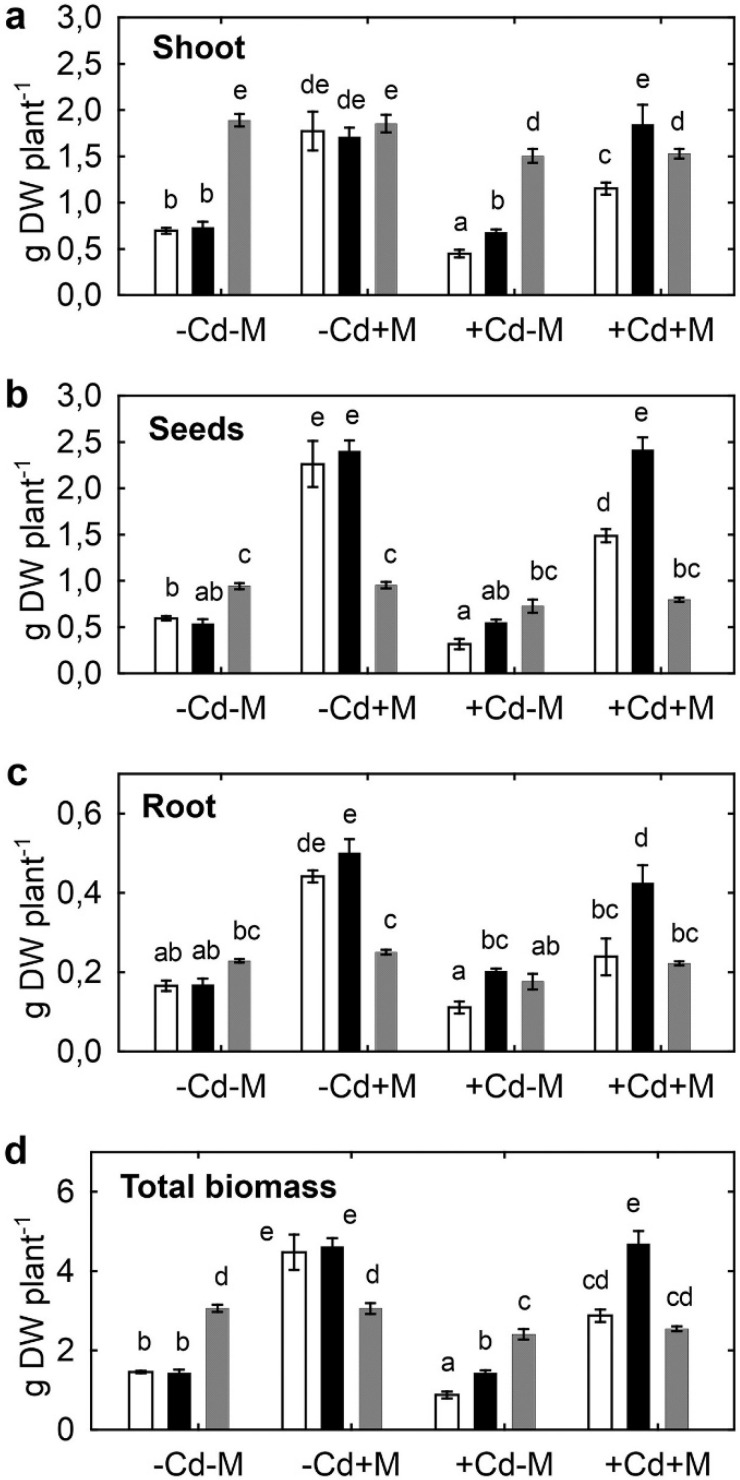
Shoot (**a**), seeds (**b**), root (**c**), and total (**d**) biomass of pea SGE (unfilled columns), SGECd^t^ (filled columns), and *B. juncea* (gray columns) grown in uncontaminated or Cd-supplemented soil. Treatments: -Cd-M—uncontaminated soil with uninoculated plants, -Cd+M—uncontaminated soil with inoculated plants, +Cd-M—Cd-supplemented soil with uninoculated plants, +Cd+M—Cd-supplemented soil with inoculated plants. Plants were inoculated with a microbial consortium consisting of *Variovorax paradoxus* 5C-2, *Rhizobium leguminosarum* bv. *viciae* RCAM1066, and *Glomus* sp. 1Fo. Vertical bars show standard errors. Different letters show significant differences between treatments (least significant difference test, *p* < 0.05, *n* = 20). DW stands for dry weight. Plants were analyzed on the 83rd day after planting.

**Figure 2:**
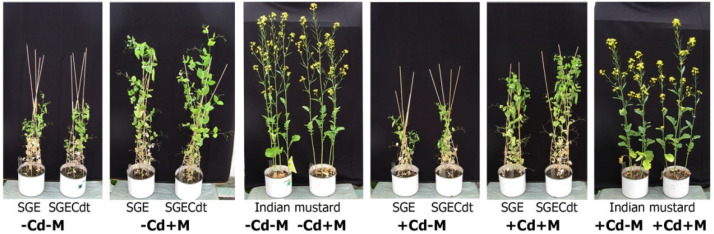
Representative plants of SGE, SGECd^t^, and Indian mustard (*B. juncea*) photographed on the 42nd day after planting. Treatments: -Cd-M—uncontaminated soil with uninoculated plants, -Cd+M—uncontaminated soil with inoculated plants, +Cd-M—Cd-supplemented soil with uninoculated plants, +Cd+M—Cd-supplemented soil with inoculated plants. Plants were inoculated with a microbial consortium consisting of *Variovorax paradoxus* 5C-2, *Rhizobium leguminosarum* bv. *viciae* RCAM1066, and *Glomus* sp. 1Fo.

Cadmium significantly inhibited shoot, seed, and total biomass production of uninoculated and particularly inoculated SGE plants (Figure 1a,b,d). Root growth of inoculated SGE plants was also inhibited by Cd and a corresponding tendency was observed for uninoculated SGE roots (*p* = 0.025; *n* = 5; Student’s *t* test). Contrary to this, biomass production of the SGECd^t^ mutant was not affected by Cd treatment, except for decreased root biomass (by 15%) of inoculated plants (Figure 1c). Inoculation with symbiotic microorganisms significantly (by 2–3 times, *p* < 0.01) increased biomass of all plant organs of both pea genotypes in the absence and presence of toxic Cd concentration (Figure 1). Growth of *B. juncea* was less affected by Cd than SGE, with significant negative effects on shoot biomass of uninoculated and inoculated plants and on total biomass of uninoculated plants (Figure 1a). Inoculation with microorganisms did not affect *B. juncea* biomass (Figure 1). Representative plants of pea genotypes and *B. juncea* photographed at 42 DAP are presented in Figure 2.

### 2.2. Symbiotic Activities of Microorganisms and Plants

When the pea genotypes were grown in uncontaminated soil, there were no genotypic differences in the symbiotic parameters measured (Table 1). At 14 DAP, the number of *V. paradoxus* 5C-2 on pea roots was about three times bigger than on *B. juncea* roots. Over the course of the experiment, this difference became insignificant and at 83 DAP the number of *V. paradoxus* 5C-2 decreased about 10 times independently of soil Cd concentration (Table 1). Treatment with Cd decreased nodule number and acetylene reduction activity of SGE by 5.6 and 10.8 times, respectively, whereas the decrease in these parameters in SGECd^t^ roots was 2.1 and 2.8 times only (Table 1). Frequency of mycorrhizal structures decreased in roots of Cd-treated SGE by 25%. Significant difference (*p* = 0.02) between SGE and SGECd^t^ grown in Cd-supplemented soil was observed for relative arbuscular richness in roots (Table 1). *B. juncea* did not form nodules or mycorrhiza, as expected.

### 2.3. Cadmium Concentration in Plants

**Figure 3:**
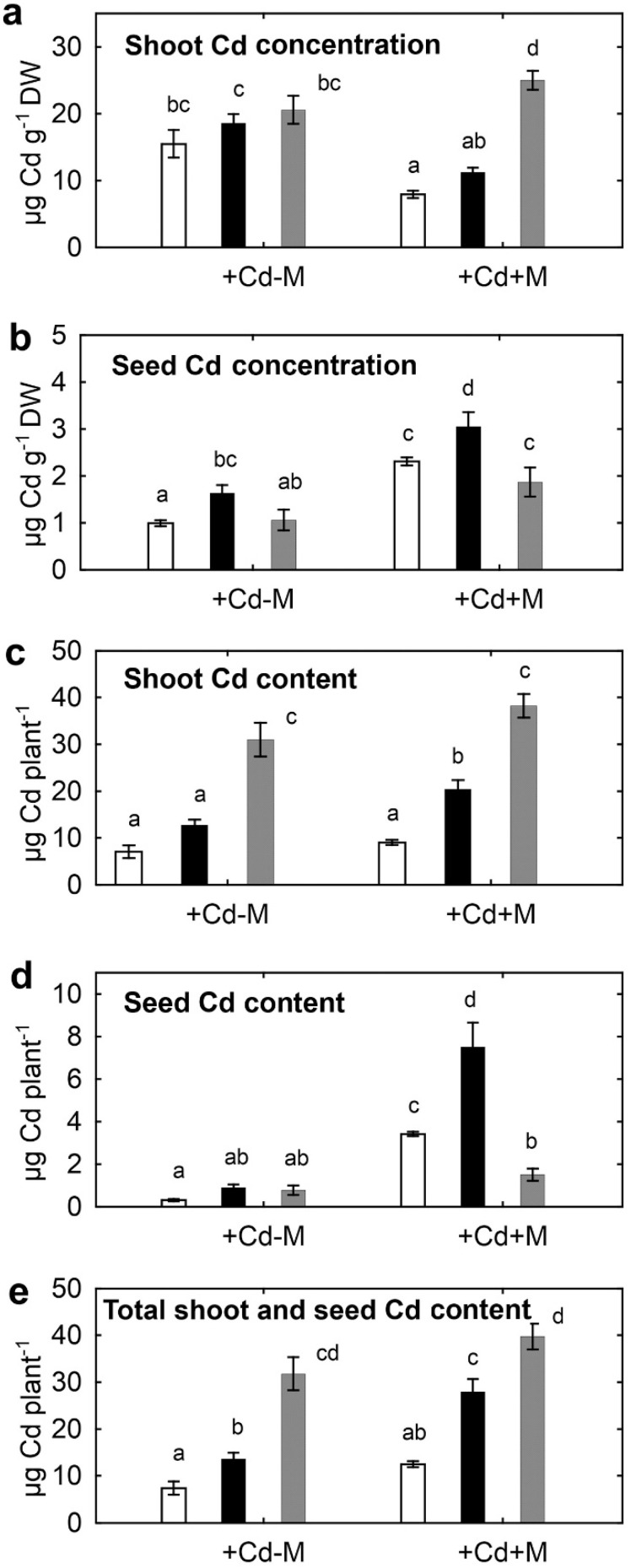
Cadmium concentration in shoot (**a**) and seeds (**b**), and Cd content in shoot (**c**), seeds (**d**) and aerial part (**e**) of pea SGE (unfilled columns), SGECd^t^ (filled columns) and *B. juncea* (gray columns) grown in Cd-supplemented soil. Treatments: +Cd-M—Cd-supplemented soil with uninoculated plants, +Cd+M—Cd-supplemented soil with inoculated plants. Plants were inoculated with a microbial consortium consisting of *Variovorax paradoxus* 5C-2, *Rhizobium leguminosarum* bv. *viciae* RCAM1066, and *Glomus* sp. 1Fo. Vertical bars show standard errors. Different letters show significant differences between treatments (least significant difference test, *p* < 0.05, *n* = 5). DW stands for dry weight. Plants were analyzed on the 83rd day after planting.

Inoculation decreased shoot Cd concentration of both pea genotypes but increased shoot Cd concentration of *B. juncea* (Figure 3a). On the contrary, Cd concentration in pea seeds increased due to inoculation (Figure 3b). The mutant seeds had a higher Cd concentration compared to wild-type SGE, and this trend was observed for shoots of inoculated plants (*p* = 0.005; *n* = 5; Student’s *t* test). *B. juncea* had a maximal value of shoot Cd concentration, whereas mutant SGECd^t^ possessed maximal Cd concentration in seeds, particularly in inoculated plants. Accumulation of Cd (Cd content) in *B. juncea* shoots was about three and two times more than in shoots of SGE and SGECd^t^, respectively (Figure 3c). Inoculation increased content Cd in pea seeds by about 10 times with a maximal value for SGECd^t^, but scarcely affected Cd content in *B. juncea* seeds (Figure 3d). As a result, Cd content in the aboveground part of SGECd^t^ was twice that of SGE, and the inoculated SGECd^t^ had approximately similar Cd accumulation capacity as compared with uninoculated *B. juncea* (Figure 3e).

### 2.4. Plant Nutrient Uptake

Both pea genotypes grown in Cd-supplemented soil had lower N concentration, N content, and ^15^N content in shoots of inoculated plants than the inoculated plants grown in uncontaminated soil (Table 2). Nevertheless, the Cd-treated and inoculated mutant SGECd^t^ had higher shoot N content and ^15^N content than SGE. In addition, shoot N concentration of the mutant was greater after inoculation of Cd-untreated plants and in uninoculated Cd-treated plants. Cadmium treatment decreased N and ^15^N content in seeds of inoculated wild-type SGE, leading to significant genotypic differences against SGECd^t^ (Table 2). *B. juncea* shoots and seeds showed higher values of ^15^N fraction as compared with pea, suggesting better assimilation of mineral nitrogen from fertilizer by this plant species (Table 2). However, N concentration and N content were generally lower in *B. juncea* shoots and seeds. 

**Figure 4:**
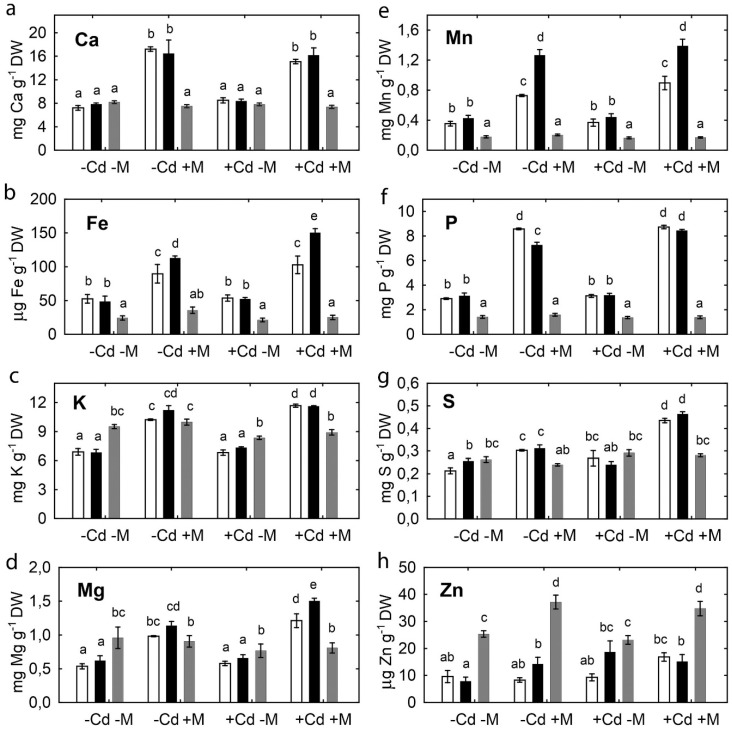
Calcium (**a**), iron (**b**), potassium (**c**), magnesium (**d**), manganese (**e**), phosphorus (**f**), sulfur (**g**), and zinc (**h**) in shoots of pea SGE (unfilled columns), SGECd^t^ (filled columns), and *B. juncea* (gray columns) grown in uncontaminated or Cd-supplemented soil. Treatments: -Cd-M—uncontaminated soil with uninoculated plants, -Cd+M—uncontaminated soil with inoculated plants, +Cd-M—Cd-supplemented soil with uninoculated plants, +Cd+M—Cd-supplemented soil with inoculated plants. Plants were inoculated with a microbial consortium consisting of *Variovorax paradoxus* 5C-2, *Rhizobium leguminosarum* bv. *viciae* RCAM1066, and *Glomus* sp. 1Fo. Vertical bars show standard errors. Different letters show significant differences between treatments (least significant difference test, *p* < 0.05, *n* = 5). DW stands for dry weight. Plants were analyzed at the 83-rd day after planting.

Inoculation with the microbial consortium significantly increased the concentration of many other nutrient elements in pea shoots (Figure 4). This effect varied according to pea genotype and soil Cd status. The most pronounced effect of inoculation was observed on Ca, Fe, Mg, Mn, and P concentrations. Cadmium treatment had no effect on shoot nutrient concentrations of uninoculated plants, except increased S concentration in SGE (Figure 4g) and Zn concentration in SGECd^t^ (Figure 4h). In inoculated plants, the Cd treatment increased Fe, Mg, P, and S concentrations in SGECd^t^ shoots, and K, Mg, and S concentrations in SGE shoots, respectively. Inoculation also induced genotypic differences, with higher Fe, Mg, and Mn concentrations in the inoculated SGECd^t^ (Figure 4b,d,e) than wild-type (WT) plants. In contrast to their effect on pea plants, inoculation and Cd treatment did not affect elemental concentration of *B. juncea* shoots (Figure 4).

**Figure 5:**
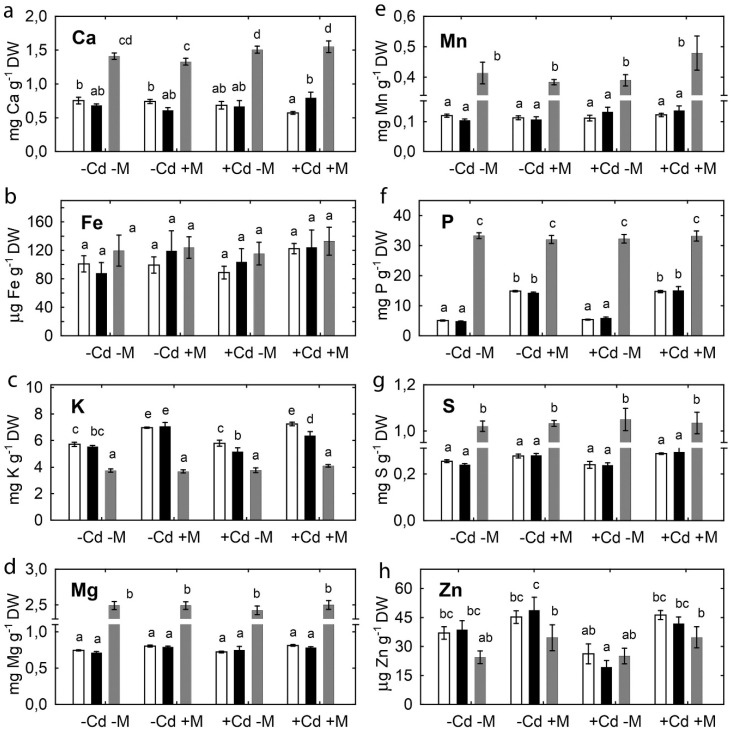
Calcium (**a**), iron (**b**), potassium (**c**), magnesium (**d**), manganese (**e**), phosphorus (**f**), sulfur (**g**), and zinc (**h**) in seeds of pea SGE (unfilled columns), SGECd^t^ (filled columns), and *B. juncea* (gray columns) grown in uncontaminated or Cd-supplemented soil. Treatments: -Cd-M—uncontaminated soil with uninoculated plants, -Cd+M—uncontaminated soil with inoculated plants, +Cd-M—Cd-supplemented soil with uninoculated plants, +Cd+M—Cd-supplemented soil with inoculated plants. Plants were inoculated with a microbial consortium consisting of *Variovorax paradoxus* 5C-2, *Rhizobium leguminosarum* bv. *viciae* RCAM1066, and *Glomus* sp. 1Fo. Vertical bars show standard errors. Different letters show significant differences between treatments (least significant difference test, *p* < 0.05, *n* = 5). DW stands for dry weight. Plants were analyzed on the 83rd day after planting.

Generally, inoculation did not affect seed nutrient concentration except for increased K and P concentration in both pea genotypes grown in both soils, and increased Zn concentration in Cd-treated SGECd^t^ (Figure 5). Cadmium treatment also had little effect on seed nutrient concentrations except for decreased Ca concentration in inoculated SGE, decreased K concentration in inoculated SGECd^t^, and decreased Zn concentration in uninoculated SGECd^t^. Pea genotype had no effect on seed nutrient concentrations, with the only significant differences being the higher Ca concentration (Figure 5a) and the lower K concentration (Figure 5c) in inoculated and uninoculated SGECd^t^, respectively.

Inoculated pea plants accumulated several times more nutrients in shoots (Table S1) and seeds (Table S2) than uninoculated plants. This occurred for both pea genotypes, but not for *B. juncea*. The SGECd^t^ mutant accumulated about twice as many nutrients in shoots and seeds than SGE, when pea plants were grown in Cd-supplemented soil and inoculated with the microbial consortium. A similar tendency was observed for Cd-treated uninoculated pea plants if genotypic difference was estimated by Student’s *t* test (*p* < 0.05; *n* = 5; Student’s *t* test). Treatment with Cd decreased Ca, Fe, K, and P contents in shoots (Table S1) and Ca, K, Mg, P, S, and Zn contents in seeds (Table S2) of SGE. However, Cd treatment did not affect nutrient accumulation in the uninoculated SGECd^t^ mutant, and even increased shoot nutrient status of inoculated plants, e.g., Fe, Mg, Mn, P, and S (Table S1). Also, Cd treatment decreased K, Mg, Mn, P, S, and Zn concentration in shoots and/or seeds of *B. juncea* (Tables S1 and S2).

## 3. Discussion

### 3.1. Plant Growth

The Cd-tolerant SGECd^t^ mutant grew better than the WT when exposed to Cd, as expected and consistent with previous observations when these genotypes were grown in Cd-supplemented soil and inoculated with *R. leguminosarum* bv. *viciae* RCAM1066 [39]. Here inoculation with the microbial consortium significantly increased biomass of both pea genotypes, but particularly of the SGECd^t^ mutant grown in Cd-supplemented soil, suggesting establishment of efficient symbiotic interactions under stressful conditions. This was most probably due to initial specific selection of the introduced strains *V. paradoxus* 5C-2 [40], nodule bacterium *R. leguminosarum* bv. *viciae* RCAM1066 [41], and *Glomus* sp. 1Fo [42] as Cd-tolerant and efficient micro-symbionts.

Inoculating with symbiotic microorganisms promoted growth and nutrition of various legume plants grown in HM-contaminated soils [9,17,18]. Pea plants grown in Cd-contaminated soil had bigger shoot and root biomass after inoculation with PGPR *Pseudomonas brassicacearum* Am3 and *P. marginalis* Dp1 having ACC deaminase activity [43]. ACC-utilizing PGPR *V. paradoxus* 5C-2 stimulated growth and development nitrogen-fixing symbiosis in pea mutant SGECd^t^ in the presence of toxic Cd in quartz sand culture [38]. Inoculation with various Cd-tolerant *R. leguminosarum* bv. *viciae* strains increased biomass and accumulation of N in Cd-treated pea varieties [44]. The important role of rhizobia and their ACC deaminase activity in alleviation of Cd stress for pea plants was recently demonstrated using *R. leguminosarum* bv. *viciae* RCAM1066 and the SGECd^t^ mutant [39]. Inoculating pea plants with the AMF *Glomus intraradices* BEG141 attenuated the negative effects of toxic soil Cd on pea growth and leaf chlorophyll fluorescence due to sequestration of Cd in roots [45]. Similarly, *G. intraradices* BEG141 stimulated biomass of the same pea genotypes in another experiment with Cd-contaminated soil; however, additional inoculation with *R. leguminosarum* bv. *viciae* strain G and/or with ACC-utilizing PGPR *P. brassicacearum* Am3 had no further effects [46]. Thus, successful application of microbial consortia requires active interaction between the microbial components and the plant.

Cd tolerance of *B. juncea* was higher than of pea SGE, as estimated by percentage of Cd-induced decrease in biomass production (Figure 1). However, the microbial consortium counteracted the negative effect of Cd on SGE, thus stimulating biomass production even of uninoculated plants grown in uncontaminated soil. On the contrary, the microorganisms had little effect on *B. juncea* growth, as it cannot form symbiosis with rhizobia and AMF. *R. leguminosarum* bv. *viciae* RCAM1066 together with *Glomus* sp. 1Fo seemed to play a crucial role in pea growth promotion, while *V. paradoxus* 5C-2 was insufficient to stimulate growth of *B. juncea*. Positive effects of *V. paradoxus* 5C-2 on pea were likely caused by stimulating nitrogen-fixing symbiosis via ACC deaminase activity. Indeed, an ACC deaminase mutant of this strain had limited effects on nodulation in pea plants subjected to drought [47], and wild-type *V. paradoxus* 5C-2 restored nodulation of Cd-treated SGE and SGECd^t^ after inoculation with *R. leguminosarum* bv. *viciae* RCAM1066 [38].

Applying microbial consortia of PGPR, rhizobia, and AMF showed additive positive effects on growth and nutrition of other legume plants: (1) *Medicago sativa* [48] and (2) *M. arborea* [49] inoculated with *Enterobacter* sp., *Sinorhizobium meliloti*, and *G. mosseae* and grown in uncontaminated soil; (3) *Trigonella foenum-graecum* inoculated with ACC-utilizing *Bacillus subtilis*, *S. meliloti*, and *Rhizophagus irregularis* and grown in arid soil [50]. In addition, Barnawal et al. [51] presented similar positive results on inoculation of pea with ACC-utilizing *Arthrobacter protophormiae*, *R. leguminosarum* bv. *Viciae*, and *G. mosseae* and subjected to salinity stress. These reports support the view that microbial consortia can increase adaptation of plants to adverse soil factors, including plants subjected to HM toxicity, more efficiently than their components.

### 3.2. Assessing Microbial Symbioses

Strain *V. paradoxus* 5C-2 was initially isolated from the rhizosphere of *B. juncea* cultivated in Cd-contaminated soil and possessed high Cd tolerance, produced auxins, and showed ACC deaminase activity [40]. Here, *V. paradoxus* 5C-2 colonized the roots of both pea genotypes grown in Cd-contaminated soil even more actively than *B. juncea* roots, and the number of bacteria was comparable to our previous experiments with pea cultivar Sparkle grown in uncontaminated soil [47]. This indicates that the pea rhizosphere is a favorable ecological niche for this strain. Therefore, it is logical to assume its active association with pea roots can be expressed in positive effects on plant growth and nodule formation [38,47]. Plant growth promotion by *V. paradoxus* 5C-2 might be related to production of auxins and ACC deaminase activity, since these traits are considered as the main mechanisms for stimulating plant growth and promoting nitrogen-fixing symbiosis by PGPR under stressful conditions, including HM toxicity [14,17,20].

It was assumed that symbiosis between legumes and rhizobia is sensitive to HMs, thus creating difficulties in using such plant–microbe systems for phytoremediation [9,52]. Therefore genetic engineering of HM-tolerant plants was proposed as a promising approach to solve this problem [15]. High Cd toxicity for nodulation of pea was also shown [39,53]. Our previous experiments with pea showed that the plant is more sensitive to Cd than rhizobia [41]. Inhibition of growth, uptake of nutrients, and induction of oxidative stress in pea plants caused by Cd was repeatedly demonstrated [53,54,55,56]. At the same time, many isolated *R. leguminosarum* bv. *viciae* strains, including RCAM1066, were described as Cd-tolerant [41,44]. These findings align with the results obtained here demonstrating the ability of Cd-tolerant pea genotype to form efficient nitrogen-fixing symbiosis with Cd-tolerant rhizobia in the presence of toxic Cd. Previously, the ACC deaminase of *R. leguminosarum* bv. *viciae* RCAM1066 counteracted the negative effects of Cd on nodulation of SGECd^t^ [39]. Rhizobial ACC deaminase plays important role in adaptation of legumes to abiotic stresses [21,24], including HM toxicity [57]. In our experiments a combination of ACC deaminase activities of rhizobia and PGPR may contribute to the formation of symbiosis and growth stimulation of the SGECd^t^ mutant.

The important role of AMF in the adaptation of various plants to unfavorable environmental conditions and in the phytoremediation of HM-polluted soils is well documented [12,58]. Because many AMF are highly resistant to HMs, mycorrhizal associations form in toxic soils [10,13]. In our experiments *Glomus* sp. 1Fo was able to form mycorrhiza on pea roots and Cd had little effect on formation of mycorrhizal structures even on relatively Cd-sensitive genotype SGE. This suggests successful development and function of this symbiosis under Cd stress.

It is important to emphasize that PGPR, rhizobia, and AMF possess functional compatibility and can exert positive effects on each other’s development [22,23,59]. For example, increased nodulation and mycorrhization was observed on chickpea (*Cicer arietinum* L.) inoculated with both *Mesorhizobium ciceri* and *Glomus etunicatum* [60]. Inoculation with *P. putida* and an unidentified AMF stimulated nodulation of subterranean clover (*Trifolium subterraneum* L.) with indigenous rhizobia [61]. Positive interactions between microorganisms introduced as triple consortia were also described. Namely: (1) *M. sativa* [48] and *M. arborea* [49] inoculated with *Enterobacter* sp., *S. meliloti*, and *G. mosseae*; (2) *Trigonella foenum-graecum* inoculated with *B. subtilis*, *S. meliloti*, and *R. irregularis* [50]; (3) pea inoculated with *A. protophormiae*, *R. leguminosarum* bv. *Viciae*, and *G. mosseae* [51]. Whether the introduced microorganisms interacted with each other could not be assessed in the present experiment since only the microbial consortium was applied. Such interactions are assumed since *V. paradoxus* 5C-2 stimulated nodulation of pea cv. Sparkle with indigenous rhizobia under water stress [47] and of mutant SGECd^t^ with *R. leguminosarum* bv. *viciae* RCAM1066 in the presence of toxic Cd [38].

### 3.3. Plant Cadmium Concentration and Accumulation

Decreased shoot Cd concentration of the inoculated pea SGE and SGECd^t^ can be explained by a dilution effect due to microbial stimulation of shoot growth, along with a limited amount of supplied Cd in pots. In accordance with this assumption, inoculation did not affect *B. juncea* shoot biomass but shoot Cd concentration slightly increased (Figure 3a). Increased shoot Cd concentration caused by inoculation of *B. juncea* with PGPR *Bacillus megaterium* was previously reported [62]. Shoots of *B. juncea* plants inoculated with ACC-utilizing and IAA-producing *Bacillus* sp. increased accumulation of Ni [63]. In contrast to shoots, inoculation increased Cd concentration in seeds of both pea and *B. juncea*, suggesting that microorganisms stimulated Cd translocation from roots into shoots and further into seeds. *V. paradoxus* 5C-2 seems to play a crucial role in this phenomenon, since this effect occurred in both plant species. The increased translocation of Cd from shoots to seeds was previously observed in pea plants inoculated with ACC-utilizing PGPR *P. brassicacearum* [46]. However, the effect of PGPR on translocation of Cd from shoots to seeds of *B. juncea* was not studied. Accepting this hypothesis, it can be assumed that *V. paradoxus* 5C-2 improved Cd tolerance mechanisms of plants via ACC deaminase activity and/or production of auxins, allowing plant tissues to tolerate Cd. Although the strain studied here, *R. leguminosarum* bv. *viciae* RCAM1066, contains ACC deaminase, the role of this trait in accumulation of Cd by SGE and SGECd^t^ was insignificant, since similar effects were observed for RCAM1066 and its ACC deaminase minus mutant [39].

Analysis of literature showed that the effects of symbiotic microorganisms on accumulation of HMs significantly varied from positive to negative, suggesting opposing approaches to using microbes for phytoremediation technologies, both phytoextraction and phytostabilization. This is relevant for various symbionts such as PGPR [15,16,17,20,64], rhizobia [8,9,10,18], and AMF [10,12,13]. Legume plants inoculated with rhizobia or AMF often had decreased HM concentrations since enhanced plant biomass diluted the effect of HM uptake; however, the effect of PGPR on shoot HM concentration in legumes was more often neutral or positive [18]. Moreover, microorganisms, particularly PGPR [65,66] and AMF [10,13], can increase or decrease mobility and availability of HMs for plants, with the resultant effect depending on many factors, such as type and properties of microorganism, plant genotype, and soil HM concentration. Interestingly, when SGE and SGECd^t^ were grown in heavily contaminated soil (30 mg Cd kg^−1^) shoot biomass halved, but inoculation with *R. leguminosarum* bv. *viciae* RCAM1066 increased shoot Cd concentration, particularly in the SGECd^t^ mutant [39]. Comparing that report with the present results also indicates that the components of the microbial consortium used here could have the opposite effects on plant Cd accumulation, and most likely that strain *Glomus* sp. 1Fo affected the decrease in shoot Cd concentration.

Shoot Cd content of inoculated pea plants was increased even as shoot Cd concentration decreased (Figure 3c). Cadmium content in pea seeds was also increased, particularly in the SGECd^t^ mutant (Figure 3d). Moreover, accumulation of Cd in the aerial part of SGECd^t^ was similar to that of the uninoculated *B. juncea* (Figure 3e), the plant considered as one of the most efficient species for HM phytoextraction technologies [1,2,3]. Inoculation with the microbial consortium and the high Cd tolerance of the SGECd^t^ mutant allowed high biomass accumulation. In addition, positive interactions between the introduced microorganisms might alter Cd accumulation. Examples confirming this hypothesis can be synergistic effects on growth and Cd accumulation by *Lotus edulis* and *L. ornithopodioides* co-inoculated with nodule bacterium *M. loti* and PGPR *V. paradoxus* 5C-2 [28], and by *T. repens* co-inoculated with AMF *G. mosseae* and PGPR *B. brevis* [25]. However, comparisons with metal-accumulating plant species like *B. juncea* were not performed in previous studies. Creating a symbiotic system comprising a Cd-tolerant legume plant and microorganisms allowed Cd phytoextraction that was as effective as using metal accumulators. Our result is an original contribution to the view that the high symbiotic potential allows legumes to be promising for phytoremediating polluted soils [9,15,18,21].

### 3.4. Plant Nutrient Uptake

The microbial consortium significantly improved N nutrition of pea plants subjected to Cd stress (Table 2) by forming a symbiosis with *R. leguminosarum* bv. *viciae* RCAM1066 that contributed to fixation of atmosphere N_2_. This also indirectly confirms the low percentage of ^15^N in pea plants compared to *B. juncea*, which does not form a symbiosis with rhizobia. On the other hand, the increased ^15^N content in inoculated pea plants grown in Cd-supplemented soil suggested better assimilation of mineral N originated from fertilizer. AMF play an important role in the uptake of mineral N from soil and fertilizers by plants [67]. Increased N uptake by pea plants inoculated with *V. paradoxus* 5C-2 under salinity [68] or with other ACC-utilizing and IAA-producing PGPR under Cd stress [43] was also reported. Therefore we propose that both *Glomus* sp. 1Fo and *V. paradoxus* 5C-2 can enhance mineral N nutrition of Cd-treated pea. Cadmium decreased nitrogen nutrition of inoculated plants when both N sources (N_2_ fixation and uptake of N fertilizer) became less available. This observation confirms the view that plant–microbe symbiosis is HM-sensitive [9,13,15]. Interestingly, significant genotypic differences in ^15^N content in shoots and seeds of SGECd^t^ demonstrated that the mutant better assimilates mineral N under stressful conditions.

That inoculation increased biomass and concentration of many nutrients in pea shoots suggested that growth of uninoculated plants was constrained by a lack of mineral nutrition. Nutrients uptake from Cd-supplemented soil by pea shoots and seeds was significantly higher in SGECd^t^ than SGE (Tables S1 and S2), suggesting better adaptation of the mutant to Cd toxicity. Indeed, maintaining nutrient homeostasis is an important mechanism of the Cd tolerance of this mutant [35]. AMF [10,13] and PGPR [16,17,23] supply plants with P and other nutrient elements from arid and HM-contaminated soils. Particularly, inoculation with ACC-utilizing and IAA-producing PGPR *V. paradoxus* 5C-2 [68] or *P. brassicacearum* Am3 and *P. marginalis* Dp1 [43] improved uptake of P, K, Ca, Mg, and Fe by pea plants treated with Cd or NaCl, respectively. The increased uptake of nutrients by the inoculated and Cd-treated SGECd^t^ agrees with the better formation of mycorrhizal structures in the Cd-treated mutant than in SGE (Table 1). Therefore, we propose that *Glomus* sp. 1Fo and *V. paradoxus* 5C-2 contributed together and probably complemented each other by improving uptake of mineral nutrients by pea, particularly by the Cd-tolerant SGECd^t^ mutant. It might be also proposed that *Glomus* sp. 1Fo was more efficient in stimulating nutrient uptake in pea as compared with *V. paradoxus* 5C-2, since the effect of the microbial consortium on *B. juncea*, which does not form symbiosis with AMF but does with PGPR, was for the most part insignificant.

## 4. Materials and Methods

### 4.1. Plants and Microorganisms

Two pea (*Pisum sativum* L.) genotypes, namely the wild-type laboratory line SGE and its Cd-tolerant mutant SGECd^t^ described previously [34], were used. Pea seeds were propagated by the authors. Seeds of Indian mustard (*Brassica juncea* L. Czern.) genotype VIR263 having high Cd tolerance [69] were obtained from the N.I. Vavilov Institute of Plant Genetic Resources (Saint-Petersburg, Russian Federation). Cadmium-tolerant strains of PGPR *V. paradoxus* strain 5C-2 [40], nodule bacterium *R. leguminosarum* bv. *viciae* strain RCAM1066 [41], and AMF strain *Glomus* sp. 1Fo [42] were obtained from the Russian Collection of Agricultural Microorganisms (RCAM, St.-Petersburg, Russian Federation, http://www.arriam.ru/kollekciya-kul-tur1/). During the experimental work the bacteria were maintained on agar yeast extract mannitol (YM) agar [70]. Inoculum *Glomus* sp. 1Fo was obtained by growing mycorrhized plants of Swedish ivy (*Plectranthus australis* L.) in sterilized soil and preparing a mixture of soil and roots with a total intensity of mycorrhizal infection of 80%. A similar soil–root mixture containing no endomycorrhizal fungi was used for inoculation as a control treatment.

### 4.2. Plant Growth Conditions

A sod-podzolic light loamy soil was dried, sieved through 5 mm mesh, sterilized by autoclaving for 4 h at 120 °C, and then incubated under sterile conditions for one month at room temperature for stabilization. Then the following soil characteristics were determined using standard methods (mg kg^−1^): total C, 25,000 ± 550; total N, 1800 ± 100; nitrate N, 10 ± 1; ammonium N, 15 ± 3; available P, 81 ± 5; available K, 87 ± 7; total Cd, 0.2 ± 0.1; total exchangeable bases, 63 ± 4 mg equiv; pH_KCl_ = 4.7 ± 0.1; pH_H2O_ = 5.5 ± 0.5. Pots were filled with 2 kg soil and each pot was fertilized with nutrient solutions resulting in (mg kg^−1^): KCl, 300; MgSO_4_, 30; CaCl_2_, 20; H_3_BO_3_, 3; MnSO_4_, 3; ZnSO_4_, 3; Na_2_MoO_4_, 2. Nitrogen fertilizer was added as ^15^NH_4_^15^NO_3_ in the amount of 15 mg kg^−1^ with a final enrichment by 35 atom % ^15^N. The soil was supplemented (or not) with CdCl_2_, yielding a concentration of 15 mg Cd kg^−1^ soil. Then the soil was watered to 80% of water holding capacity (WHC) and incubated at room temperature for 10 days for stabilization.

A pot experiment was carried out in a polyethylene greenhouse with natural lighting and temperature in summer (June–August, St.-Petersburg). The average monthly temperature, humidity, and daylight hours were for June +15.2 °C, 67.3%, 18 h; for July +19.5 °C, 71.2%, 18 h; and for August +16.2 °C, 74.3%, 18 h, respectively (Gismeteo, https://www.gismeteo.ru/). Seeds of pea and *B. juncea* were selected for homogeneity of seed weight, then surface-sterilized and scarified by treatment with 98% H_2_SO_4_ for 10 min, rinsed with sterile water, and germinated on moistened filter paper (Whatman #1) in the dark at 25 °C for 3 days. Each seedling was inoculated with a mixture of microorganisms: 1 mL of *R. leguminosarum* bv. *viciae* RCAM1066 and 1 mL of *V. paradoxus* 5C-2 water suspensions containing 10^7^ cells mL^−1^ each and supplemented with 5 g of mycorrhizal inoculum immediately after sowing. Control seeds were treated with 5 g of soil–root mixture containing no endomycorrhizal fungi. Seven pots with four uniform seedlings per pot were prepared for each plant genotype and treatment. Pots were watered up to 70% WHC with evapotranspirational losses replenished every day by weighing the pots. The plants were cultivated for 83 days until maturity and harvested.

### 4.3. Parameters of Symbiotic Activities of Microorganisms and Plants

Several times (14, 42, and 83 days after planting—DAP), the roots of one pot of each treatment were used to monitor the presence of the introduced *V. paradoxus* 5C-2 on roots as described previously [71]. Briefly, the roots of four individual plants (*n* = 4) were thoroughly shaken to remove adhering soil particles and equal portions of 100 mg fresh weight (FW) without nodules were selected. Each root portion was gently washed in sterile tap water and homogenized with mortar and pestle. The homogenates were serially diluted in 10-fold steps and 50 µL aliquots were plated in two replicates on BPF agar medium supplemented with antibiotics (30 µg mL^−1^ kanamycin and 20 µg mL^−1^ rifampicin) to which *V. paradoxus* 5C-2 is resistant. The BPF medium was additionally supplemented with 40 µg mL^−1^ nystatin to prevent fungal growth. Characteristic yellow colonies of *V. paradoxus* 5C-2, which were absent on plates containing homogenates of uninoculated roots, were counted after incubation at 30 °C for four days. Data were expressed as the number of colony forming units (CFUs) per g of root fresh weight (FW).

At 42 DAP, the remaining root tissues of each plant, together with the attached nodules, were placed separately in hermetically sealed glass flasks (four flasks per treatment, *n* = 4) and incubated for 1 h with 10% acetylene in a gas phase. The reaction was terminated by addition of Nessler’s reagent to the flasks. The nitrogen fixation activity was measured by the acetylene-reduction method [72] using a gas chromatograph GC-2014 (Shimadzu, Kyoto, Japan). After that, the roots were removed from the flasks, washed, and used for calculation of nodules.

At the end of the experiment (83 DAP) the roots of four plants from each of the remaining five pots were combined (*n* = 5), washed, dried, weighed, and prepared for estimation of mycorrhizal colonization as described by Turnau et al. [58] with some modifications. The roots were treated with 10% KOH for 10 min at 95 °C and washed with water three times. Then, the mycelium in the roots was stained by treatment for 3 min in 10% acetic acid supplemented with 5% black mascara solution (Sheaffer Pen, Shelton, Connecticut, USA) and thoroughly washed with water. Microscopy of roots was performed using light microscope Axio Lab.A1 (Carl Zeiss, Oberkochen, Germany). Frequency of mycorrhizal structures (F), colonization intensity within mycorrhizal roots (M), relative arbuscular richness (A), and relative vesicular richness (V) were assessed as described previously [73].

### 4.4. Cadmium and Nutrient Concentrations in Plants

The dried plant shoots (leaves, stems, and pod walls) and seeds were ground to a powder. Nitrogen concentration and atom % ^15^N in the ground samples were determined using the elemental analyzer (FlashEA 1112, Thermo Scientific, Italy) coupled with the isotope ratio mass spectrometer Delta V Advantage (Thermo Fisher Scientific, Dreieich, Germany) and the continuous flow interface ConFlo III following manufacturer’s instructions. To determine Cd and nutrient (Ca, Fe, K, Mg, Mn, S, P, and Zn) concentrations, the ground shoot samples were digested in a mixture of concentrated HNO_3_ and 38% H_2_O_2_ at 70 °C using DigiBlock digester (LabTech, Sorisole, Italy). Elemental concentrations of digested plant samples were determined using an inductively coupled plasma emission spectrometer ICPE-9000 (Shimadzu, Kyoto, Japan).

### 4.5. Statistical Analysis

Statistical analysis of the data was performed using the software STATISTICA version 10 (TIBCO Software Inc., Carlsbad, CA, USA). ANOVA analysis with Fisher’s LSD test and Student’s *t* test was used to evaluate differences between means.

## 5. Conclusions

A microbial consortium comprising types of micro-symbionts (PGPR, rhizobia, and AMF) stimulated growth and Cd accumulation by legume plants grown in Cd-contaminated soil. Using a specific legume–microbe system based on an HM-tolerant plant and selected microbes with high Cd tolerance and symbiotic efficiency was original: pea mutant SGECd^t^ accompanied with *V. paradoxus* 5C-2, *R. leguminosarum* bv. *viciae* RCAM1066, and *Glomus* sp. 1Fo. This system showed high symbiotic potential under stress conditions caused by Cd toxicity, allowing Cd tolerance and accumulation similar to the cruciferous plant Indian mustard (*B. juncea*) that is often applied for phytoremediation of HM-contaminated soils. The results emphasize that legume plants can be used for phytoremediation when accompanied by a consortium of symbiotic microorganisms successfully integrating with the HM-tolerant plant genotype.

## Figures and Tables

**Table 1:** Parameters of symbiotic activities of the plants inoculated with a microbial consortium and grown in uncontaminated or Cd-supplemented soil.

Treatments	Nodule Number Per Plant	Acetylene Reduction Activity (Nmol C_2_H_4_ plant^−1^ h^−1^)	Mycorrhizal Structures in Roots (%)				Number of *V. paradoxus* 5C-2 (10^6^ CFU g^−1^ Root FW)		
			F	M	A	V	14 DAP	42 DAP	83 DAP
Uncontaminated soil with inoculation									
SGE	68 ± 4 ^c^	592 ± 36 ^c^	81 ± 8 ^b^	36 ± 13 ^ab^	7 ± 2 ^ab^	22 ± 8 ^a^	9.7 ± 1.2 ^b^	4.0 ± 2.0 ^ab^	0.7 ± 0.2 ^a^
SGECd^t^	81 ± 12 ^c^	673 ± 61 ^c^	90 ± 5 ^b^	48 ± 5 ^b^	11 ± 3 ^ab^	36 ± 7 ^a^	8.7 ± 1.2 ^b^	4.3 ± 0.9 ^b^	1.1 ± 0.4 ^a^
*B. juncea*	NF	ND	NF	NF	NF	NF	3.7 ± 0.9 ^a^	1.2 ± 0.4 ^ab^	0.5 ± 0.2 ^a^
Soil supplemented with 15 mg Cd kg^−1^ with inoculation									
SGE	12 ± 4 ^a^	55 ± 8 ^a^	62 ± 3 ^a^	26 ± 4 ^a^	3 ± 2 ^a^	20 ± 3 ^a^	8.0 ± 0.6 ^b^	3.3 ± 1.5 ^ab^	1.6 ± 0.7 ^a^
SGECd^t^	39 ± 7 ^b^	236 ± 17 ^b^	86 ± 5 ^b^	44 ± 6 ^ab^	16 ± 5 ^b^	26 ± 6 ^a^	8.0 ± 1.5 ^b^	3.7 ± 1.2 ^ab^	1.9 ± 0.7 ^a^
*B. juncea*	NF	ND	NF	NF	NF	NF	3.3 ± 0.3 ^a^	0.9 ± 0.6 ^a^	0.4 ± 0.3 ^a^

Plants were inoculated with a microbial consortium consisting of *Variovorax paradoxus* 5C-2, *Rhizobium leguminosarum* bv. *viciae* RCAM1066, and *Glomus* sp. 1Fo. Different superscript letters (a, b and c) show significant differences between treatments within a column (least significant difference test, *p* < 0.05, *n* varied from 4 to 5 depending on the parameter measured). Data are means ± SE. CFU stands for colony forming units. DAP stands for days after planting. FW stands for fresh weight. ND stands for not determined. NF stands for not found. Mycorrhizal structures: frequency of mycorrhizal structures (F), colonization intensity within mycorrhizal roots (M), relative arbuscular richness (A), and relative vesicular richness (V). Nodule number and acetylene reduction activity were analyzed on the 42nd day after planting. Mycorrhizal structures were analyzed on the 83rd day after planting.

**Table 2:** Concentration and content of nitrogen in shoots and seeds of plants grown in uncontaminated or Cd-supplemented soil.

Treatments	Shoots				Seeds			
	N Concentration (mg g^−1^ DW)	^15^N Fraction (%)	N Content (mg Plant^−1^)	^15^N Content (mg Plant^−1^)	N Concentration (mg g^−1^ DW)	^15^N Fraction (%)	N Content (mg Plant^−1^)	^15^N Content (mg Plant^−1^)
Uncontaminated soil without inoculation								
SGE	7.4 ± 0.2 ^ab^	1.7 ± 0.1 ^a^	5.2 ± 0.3 ^ab^	0.09 ± 0.01 ^ab^	29.9 ± 1.7 ^c^	1.9 ± 0.1 ^ab^	17.8 ± 1.5 ^a^	0.33 ± 0.04 ^ab^
SGECd^t^	8.2 ± 0.6 ^b^	2.0 ± 0.1 ^abc^	6.0 ± 0.7 ^ab^	0.12 ± 0.01 ^ab^	27.8 ± 1.4 ^c^	2.0 ± 0.1 ^ab^	14.9 ± 1.5 ^a^	0.29 ± 0.03 ^ab^
*B. juncea*	6.1 ± 0.2 ^a^	2.5 ± 0.1 ^cd^	11.6 ± 0.5 ^bc^	0.29 ± 0.02 ^bc^	17.9 ± 1.1 ^ab^	2.5 ± 0.1 ^de^	16.9 ± 1.3 ^a^	0.42 ± 0.04 ^ab^
Uncontaminated soil with inoculation								
SGE	14.6 ± 1.5 ^d^	1.8 ± 0.2 ^ab^	26.3 ± 4.6 ^de^	0.50 ± 0.12 ^de^	44.0 ± 1.7 ^d^	1.7 ± 0.2 ^a^	98.3 ± 8.6 ^c^	1.68 ± 0.28 ^d^
SGECd^t^	17.7 ± 0.6 ^e^	1.8 ± 0.1 ^ab^	30.3 ± 2.0 ^e^	0.57 ± 0.06 ^e^	44.0 ± 2.1 ^d^	1.8 ± 0.1 ^ab^	105.6 ± 7.0 ^c^	1.92 ± 0.13 ^d^
*B. juncea*	7.1 ± 0.1 ^ab^	2.6 ± 0.1 ^d^	13.2 ± 0.6 ^c^	0.34 ± 0.02 ^cd^	20.7 ± 0.4 ^b^	2.7 ± 0.1 ^e^	19.7 ± 0.9 ^a^	0.54 ± 0.02 ^b^
Soil supplemented with 15 mg Cd kg^−1^ without inoculation								
SGE	6.7 ± 0.1 ^a^	1.8 ± 0.2 ^ab^	3.0 ± 0.3 ^a^	0.06 ± 0.01 ^a^	28.9 ± 1.6 ^c^	2.0 ± 0.2 ^ab^	9.4 ± 2.0 ^a^	0.19 ± 0.05 ^a^
SGECd^t^	8.7 ± 0.8 ^b^	1.9 ± 0.2 ^ab^	5.9 ± 0.6 ^ab^	0.12 ± 0.02 ^ab^	30.8 ± 1.9 ^c^	1.8 ± 0.1 ^ab^	17.0 ± 1.7 ^a^	0.31 ± 0.03 ^ab^
*B. juncea*	5.9 ± 0.5 ^a^	2.3 ± 0.1 ^c^	8.9 ± 0.7 ^bc^	0.20 ± 0.02 ^bc^	16.4 ± 1.0 ^a^	2.2 ± 0.2 ^cd^	12.0 ± 1.6 ^a^	0.25 ± 0.03 ^ab^
Soil supplemented with 15 mg Cd kg^−1^ with inoculation								
SGE	11.4 ± 0.7 ^c^	2.1 ± 0.1 ^bc^	13.0 ± 0.4 ^c^	0.28 ± 0.01 ^c^	42.6 ± 0.4 ^d^	2.2 ± 0.1 ^bc^	63.4 ± 3.1 ^b^	1.36 ± 0.06 ^c^
SGECd^t^	12.2 ± 0.7 ^c^	1.9 ± 0.1 ^ab^	22.0 ± 1.7 ^d^	0.42 ± 0.03 ^d^	40.8 ± 0.6 ^d^	1.9 ± 0.1 ^ab^	98.5 ± 5.0 ^c^	1.88 ± 0.12 ^d^
*B. juncea*	5.6 ± 0.5 ^a^	2.1 ± 0.1 ^bc^	8.5 ± 0.9 ^b^	0.18 ± 0.01 ^bc^	16.7 ± 1.5 ^a^	2.4 ± 0.1 ^de^	13.3 ± 1.3 ^a^	0.32 ± 0.05 ^ab^

Plants were inoculated with a microbial consortium consisting of *Variovorax paradoxus* 5C-2, *Rhizobium leguminosarum* bv. *viciae* RCAM1066, and *Glomus* sp. 1Fo. Different superscript letters (a, b, c, d and e) show significant differences between treatments within a column (least significant difference test, *p* < 0.05, *n* = 5). Different letters (a, b and c) show significant differences between treatments. Data are means ± SE. Plants were analyzed on the 83rd day after planting.

## Supplementary Materials

The following are available online at https://www.mdpi.com/2223-7747/9/8/975/s1, Table S1: Nutrient contents in shoots of plants grown in uncontaminated and Cd-supplemented soil; Table S2: Nutrient contents in seeds of plants grown in uncontaminated and Cd-supplemented soil.
