# Supplementary material for: Microbial Consortium of PGPR, Rhizobia and Arbuscular Mycorrhizal Fungus Makes Pea Mutant SGECdt Comparable with Indian Mustard in Cadmium Tolerance and Accumulation
Source: Plants (Basel). 2020 Jul 31;9(8):975. doi: 10.3390/plants9080975 (PMC7464992; doi:10.3390/plants9080975)
Supplement: Supplementary file 1 [file plants-09-00975-s001.pdf]

SUPPLEMENTARY MATERIALS

Microbial consortium of PGPR, rhizobia and arbuscular mycorrhizal fungus makes pea mutant SGECd[t] comparable with Indian mustard in cadmium tolerance and accumulation

Andrey A. Belimov[a]*, Aleksander I. Shaposhnikov[a], Tatiana S. Azarova[a], Natalia M. Makarova[a], Vera I. Safronova[a], Vladimir A. Litvinskiy[b], Vladimir V. Nosikov[b], Aleksey A. Zavalin[b], Igor A. Tikhonovich[a, c]

[a] All-Russia Research Institute for Agricultural Microbiology, Podbelskogo sh. 3, Pushkin, 196608, Saint-Petersburg, Russian-Federation.
[b] Pryanishnikov Institute of Agrochemisty, Pryanishnikova str. 31A, 127434, Moscow, Russian Federation.
[c] Saint-Petersburg State University, University Embankment, 199034, Saint-Petersburg, Russian Federation.

* Corresponding author:
Andrey A. Belimov
Tel: 007 812 4761802
Fax: 007 812 4763462
E-mail: belimov@rambler.ru

Table S1. Total amount of nutrients in shoots of plants grown in uncontaminated and Cd-supplemented soil

Treatments	Ca (mg plant^{-1})	Fe (μg plant^{-1})	K (mg plant^{-1})	Mg (mg plant^{-1})	Mn (mg plant^{-1})	P (mg plant^{-1})	S (mg plant^{-1})	Zn (μg plant^{-1})
Uncontaminated soil without inoculation								
SGE	5.1 ± 0.5 a	36 ± 3 a	4.8 ± 0.3 a	0.38 ± 0.04 a	0.25 ± 0.03 a	2.0 ± 0.1 a	0.15 ± 0.01 a	7 ± 2 a
SGECd^{t}	5.7 ± 0.4 a	36 ± 8 a	4.9 ± 0.3 a	0.45 ± 0.06 a	0.32 ± 0.05 a	2.3 ± 0.2 a	0.19 ± 0.02 a	6 ± 1 a
B. juncea	15.5 ± 0.7 bc	46 ± 6 a	17.9 ± 0.3 c	1.81 ± 0.30 c	0.34 ± 0.03 a	2.7 ± 0.3 a	0.50 ± 0.04 b	48 ± 3 d
Uncontaminated soil with inoculation								
SGE	30.6 ± 4.0 d	167 ± 44 dc	18.1 ± 2.2 c	1.75 ± 0.22 c	1.30 ± 0.17 b	15.3 ± 1.9 d	0.54 ± 0.07 b	14 ± 1 ab
SGECd^{t}	27.8 ± 3.3 d	194 ± 16 c	19.2 ± 1.4 cd	1.95 ± 0.16 c	2.18 ± 0.19 c	12.5 ± 0.9 c	0.54 ± 0.04 b	25 ± 5 bc
B. juncea	13.8 ± 0.4 bc	66 ± 11 a	18.5 ± 1.1 cd	1.66 ± 0.12 c	0.38 ± 0.03 a	3.0 ± 0.3 a	0.44 ± 0.03 b	69 ± 7 e
Soil supplemented with 15 mg Cd kg^{-1} without inoculation								
SGE	3.8 ± 0.5 a	25 ± 5 a	3.1 ± 0.3 a	0.26 ± 0.03 a	0.16 ± 0.03 a	1.4 ± 0.2 a	0.12 ± 0.02 a	4 ± 1 a
SGECd^{t}	5.7 ± 0.2 a	35 ± 2 a	5.0 ± 0.2 a	0.45 ± 0.04 a	0.30 ± 0.03 a	2.2 ± 0.1 a	0.16 ± 0.01 a	13 ± 3 ab
B. juncea	11.7 ± 0.7 b	32 ± 4 a	12.5 ± 0.6 b	1.15 ± 0.15 b	0.25 ± 0.02 a	2.0 ± 0.1 a	0.44 ± 0.04 b	35 ± 4 c
Soil supplemented with 15 mg Cd kg^{-1} with inoculation								
SGE	17.4 ± 1.4 c	117 ± 13 b	13.4 ± 0.7 b	1.38 ± 0.10 dc	1.02 ± 0.09 b	10.0 ± 0.5 b	0.50 ± 0.04 b	19 ± 1 b
SGECd^{t}	29.7 ± 4.4 d	279 ± 36 d	21.5 ± 2.5 d	2.76 ± 0.30 d	2.62 ± 0.44 d	15.6 ± 1.8 d	0.85 ± 0.10 c	29 ± 7 bc
B. juncea	11.2 ± 0.4 b	38 ± 5 a	13.6 ± 0.5 b	1.24 ± 0.12 b	0.26 ± 0.02 a	2.1 ± 0.2 a	0.43 ± 0.02 b	54 ± 5 d

Plants were inoculated with microbial consortium consisting of *Variovorax paradoxus* 5C-2, *Rhizobium leguminosarum* bv. *viciae* RCAM1066 and *Glomus* sp. 1Fo. Different letters show significant differences between treatments within a column (least significant difference test, $P < 0.05$, $n = 5$).

Table S2. Total amount of nutrients in seeds of plants grown in uncontaminated and Cd-supplemented soil

Treatments	Ca (mg plant^{-1})	Fe (µg plant^{-1})	K (mg plant^{-1})	Mg (mg plant^{-1})	Mn (µg plant^{-1})	P (mg plant^{-1})	S (mg plant^{-1})	Zn (µg plant^{-1})
Uncontaminated soil without inoculation								
SGE	0.45 ± 0.04 a	60 ± 6 ab	3.4 ± 0.2 a	0.44 ± 0.02 a	72 ± 5 a	3.0 ± 0.2 a	0.15 ± 0.01 a	22 ± 3 ab
SGECd^{t}	0.36 ± 0.03 a	45 ± 5 ab	3.0 ± 0.3 a	0.38 ± 0.03 a	56 ± 4 a	2.6 ± 0.2 a	0.13 ± 0.01 a	21 ± 3 ab
B. juncea	1.33 ± 0.05 c	112 ± 20 bc	3.5 ± 0.1 a	2.35 ± 0.07 d	392 ± 43 d	31.4 ± 1.4 cd	0.96 ± 0.05 e	23 ± 3 ab
Uncontaminated soil with inoculation								
SGE	1.68 ± 0.20 d	224 ± 36 cd	15.7 ± 1.7 c	1.83 ± 0.22 c	262 ± 41 bc	33.5 ± 3.4 cd	0.62 ± 0.05 c	103 ± 14 d
SGECd^{t}	1.45 ± 0.08 cd	275 ± 54 d	16.9 ± 0.8 c	1.90 ± 0.09 c	255 ± 16 bc	34.3 ± 1.6 cd	0.67 ± 0.05 cd	114 ± 11 d
B. juncea	1.27 ± 0.07 c	118 ± 14 bc	3.5 ± 0.1 a	2.37 ± 0.05 d	365 ± 16 cd	30.5 ± 1.4 c	0.99 ± 0.04 e	33 ± 7 b
Soil supplemented with 15 mg Cd kg^{-1} without inoculation								
SGE	0.22 ± 0.04 a	27 ± 5 a	1.8 ± 0.3 a	0.23 ± 0.04 a	36 ± 7 a	1.7 ± 0.3 a	0.08 ± 0.02 a	8 ± 2 a
SGECd^{t}	0.37 ± 0.07 a	57 ± 12 ab	2.8 ± 0.3 a	0.41 ± 0.03 a	74 ± 11 a	3.3 ± 0.4 a	0.13 ± 0.01 a	11 ± 2 ab
B. juncea	1.10 ± 0.12 bc	82 ± 10 b	2.7 ± 0.3 a	1.77 ± 0.20 c	287 ± 39 c	23.7 ± 3.0 b	0.77 ± 0.10 d	18 ± 2ab
Soil supplemented with 15 mg Cd kg^{-1} with inoculation								
SGE	0.86 ± 0.07 b	182 ± 14 c	10.8 ± 0.5 b	1.21 ± 0.06 b	183 ± 11 b	21.9 ± 1.2 b	0.42 ± 0.02 b	69 ± 4 c
SGECd^{t}	1.92 ± 0.25 d	305 ± 66 d	15.3 ± 0.7 c	1.89 ± 0.10 c	336 ± 52 cd	36.9 ± 5.0 d	0.71 ± 0.08 c	101 ± 10 d
B. juncea	1.24 ± 0.09 c	106 ± 16 b	3.3 ± 0.2 a	1.99 ± 0.11 c	382 ± 45 d	26.6 ± 2.0 bc	0.83 ± 0.05 d	28 ± 4 b

Plants were inoculated with microbial consortium consisting of *Variovorax paradoxus* 5C-2, *Rhizobium leguminosarum* bv. *viciae* RCAM1066 and *Glomus* sp. 1Fo. Different letters show significant differences between treatments within a column (least significant difference test, $P < 0.05$, $n = 5$).
